# Swine Influenza A(H3N2) Virus Infection in Immunocompromised Man, Italy, 2014

**DOI:** 10.3201/eid2107.140981

**Published:** 2015-07

**Authors:** Antonio Piralla, Ana Moreno, Maria Ester Orlandi, Elena Percivalle, Chiara Chiapponi, Fausto Vezzoli, Fausto Baldanti, the Influenza Surveillance Study Group

**Affiliations:** Fondazione Istituto Di Ricovero e Cura a Carattere Scientifico Policlinico San Matteo, Pavia, Italy (A. Piralla, M.E. Orlandi, E. Percivalle, F. Baldanti);; Istituto Zooprofilattico Sperimentale della Lombardia ed Emilia Romagna, Brescia, Italy (A. Moreno);; Istituto Zooprofilattico Sperimentale della Lombardia ed Emilia Romagna, Parma, Italy (C. Chiapponi);; Istituto Zooprofilattico Sperimentale della Lombardia ed Emilia Romagna, Lodi, Italy (F. Vezzoli)

**Keywords:** Swine influenza, subtype H3N2, zoonoses, viruses, Italy, influenza

## Abstract

Because swine influenza virus infection is seldom diagnosed in humans, its frequency might be underestimated. We report a immunocompromised hematologic patient with swine influenza A(H3N2) virus in 2014 in Italy. Local pigs were the source of this human infection.

Pigs are considered the “mixing vessel” in which avian, human, and swine influenza genetic material can be exchanged and result in new influenza viruses (*1*). Zoonotic influenza A infections in humans caused by swine influenza viruses (SIVs) have been infrequently reported in Europe (*1*,*2*), even though at least 19% of occupationally exposed humans, such as pig farmers, slaughterers, and veterinarians, have SIV antibodies (*3*). However, because the infection is clinically mild in most cases, its frequency might be underdiagnosed in humans (*4*).

Three influenza A subtypes (H1N1, H1N2, and H3N2) circulate in swine herds in Italy (*1*). We report a European swine A(H3N2) influenza virus that occurred in an immunocompromised man in Italy in 2014.

## The Study

On January 14, 2014, a 67-year-old man with multiple myeloma underwent the eighth cycle of chemotherapy at the Hematology Unit of the Fondazione Istituto Di Ricovero e Cura a Carattere Scientifico Policlinico San Matteo in Pavia, Italy. The patient had mild upper respiratory syndrome (fever, cough, and cold). A nasal swab sample was tested by real-time reverse transcription PCR (RT-PCR) and PCR with a panel for 17 respiratory viruses (*5*,*6*). The clinical specimen was positive for influenza A (6 × 10^6^ RNA copies/mL). However, attempts to subtype the strain by using real-time RT-PCR specific for human influenza subtypes H1 and H3, as well as avian influenza subtype H7N9, were unsuccessful.

The clinical sample was inoculated onto a mixed-cell (Mv1Lu and A549 cells) monolayer. After 48 h incubation, it scored positive using a monoclonal antibody specific for influenza A/H3 antigen (Millipore, Billerica, MA, USA).

On January 24, 2014, the influenza virus strain A/Pavia/07/2014 was recovered from the supernatant propagated in MDCK cell culture. An RT-PCR that amplifies all 8 segments of the influenza A genome was then conducted (*7*). The purified amplicons were sequenced by using the BigDye Terminator v3.1 Cycle Sequencing Kit (Applied Biosystems, Foster City, CA, USA) in a 3130xl Genetic Analyzer (Applied Biosystems). We BLAST searched the sequences obtained for closely related sequences in GenBank (http://blast.ncbi.nlm.nih.gov/Blast.cgi). Partial nucleotide sequences of polymerase, nucleoprotein, hemagglutinin (HA), neuraminidase (NA), matrix, and nonstructural genes showed swine influenza A(H3N2) virus with an internal gene belonging to the European SIV lineage.

At a second control visit, on January 29, 2014, the patient’s nasal swab sample was negative for respiratory viruses, but cough and cold persisted. No vaccination or antiviral treatment was administered to the patient before or during the influenza episode. During January 2014, he had spent several weeks visiting his relatives on a pig farm in the province of Lodi (northern Italy) and reported contact with pigs, as well as with his grandson. No respiratory symptoms had developed in any of his family members (owners of the pig farm) or in farm co-workers.

**Figure 1:**
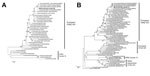
Phylogenetic trees of the hemagglutinin (A) and neuraminidase (B) genes of swine influenza viruses (SIVs). The 8 genome segment sequences of the A/Pavia/07/2014 strain (black dot, in bold) were submitted to GenBank under accession nos. KJ623706–KJ623713. Scale bars indicate nucleotide substitutions per site.

On February 6, 2014, the A/Pavia/07/2014 strain was propagated in embryonated specific pathogen–free chicken eggs at the Istituto Zooprofilattico Sperimentale della Lombardia ed Emilia Romagna (Brescia, Italy). The sequences of complete genome segments were obtained with the MiSeq platform (Illumina, San Diego, CA, USA) as previously described (*8*). The data were de novo assembled on BaseSpace Cloud (Illumina) with the DNAStar application and analyzed with the Lasergene package software (version 10.1.2). We conducted phylogenetic analysis online using PhyML v.3.0 (*9*) and MEGA5 software (*10*). A phylogenetic tree of the HA and NA genes confirmed that the A/Pavia/07/2014 strain was closely related to European A(H3N2) SIV (Figure 1). In addition, phylogenetic trees constructed with sequences of the polymerase base 1, polymerase base 2, polymerase, nucleoprotein, matrix, and nonstructural genes showed that the A/Pavia/07/2014 strain clustered within the European avian-like SIVs, including H1N1, H1N2 and H3N2 subtypes (Technical Appendix Figure 1).

**Figure 2:**
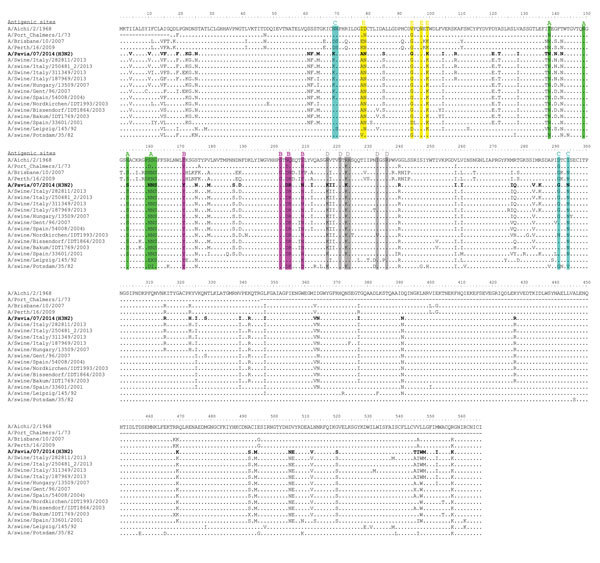
Amino acid sequence alignment of the hemagglutinin protein of swine influenza virus (SIV) (H3N2) strain A/Pavia/07/2014 (bold) and other SIVs. Antigenic sites A, B, C, D, and E of H3 HA are highlighted in green, magenta, blue, gray, and yellow, respectively, as proposed by others (*11*). Amino acid changes with respect to the A/Aichi/2/1968 strain are indicated for each strain.

The HA gene of the A/Pavia/07/2014 strain is 567 aa long and has antigenic sites identical to those of SIV A(H3N2) strains that circulated in swine in Italy during 2013 (Figure 2). In addition, the pattern of A/Pavia/07/2014 glycosylation sites in the HA is identical to that in the A/swine/Italy/282811/2013 HA sequence and different from the human influenza A/Brisbane/10/2007 strain (Technical Appendix Figure 2).

In December 2013, respiratory symptoms were observed mainly in piglets and weaning pigs on the farm that the patient visited, a farrow-to-finish pig farm where 400 sows were reared. The various production phases (mating, gestation, farrowing, nursery, and growing/finishing) were located in separated buildings. Forty-two nasal swab samples were collected at the end of January 2014 from pigs at all production phases. Considering that clinical signs were observed in piglets and weaning pigs, most samples (30 samples) were collected from weaning pigs. Influenza A real-time RT-PCR yielded negative results, probably because samples were collected ≈2 months after clinical signs appeared. Serologic investigations were performed on 29 serum samples from sows of different ages in mid-May that were collected within a national monitoring plan for Aujeszky disease. Samples were tested by hemagglutination-inhibition test according to standard procedures (*12*) by using A/Pavia/07/2014 and the reference SIVs A/swine/CA/3633/84 H3N2, A/swine/Italy/1521/98 H1N2, and A/swine/Finistere/2899/82 as antigens. Two-fold serum dilutions were tested starting at 1:20. All animals showed antibodies against H3 (137.16 geometric mean vs. A/Pavia/07/2014 and 84.2 vs. A/swine/CA/3633/84), whereas 15 of 29 serum samples that originated from the oldest animals also yielded positive results for A(H1N1) SIV. No antibodies against A(H1N2) SIV were detected.

## Conclusions

The swine influenza A(H3N2) viruses present in Europe since 1984 resulted from a genomic reassortment between human-like swine H3N2 viruses and avian-like swine H1N1 viruses (*13*). Until 2011, only 3 episodes of SIV H3N2 infection had been reported in the Netherlands and Switzerland (*1*,*2*). Recently, in 2011 the emergence of a new SIV H3N2 variant was reported in the United States that had limited person-to-person transmission (*14*). Here we report a case of SIV H3N2 infection in a human host in January 2014 in Italy. In this patient, the presence of an uncommon influenza strain was suspected after the failure of molecular typing in the presence of high influenza load. The SIV strain identified here correlated with strains circulating in pigs during the 2013–14 influenza season in Italy. In addition, serologic results on pig serum collected from a farm close to the patient’s home suggested a recent exposure with an H3N2 strain similar to the A/Pavia/07/2014 isolate. These virologic and serologic data suggest that local pigs were the source of human infection. 

In agreement with previous observations (*1*,*2*), the European H3N2 swine viruses seem to cause a benign disease with mild influenza-like symptoms in humans. In addition, the SIV strain we identified was found only in an immunocompromised patient. The uncomplicated clinical course might not be uncommon in immunocompromised patients. Indeed, in 2 epidemiologically correlated immunocompromised patients, the emergence of human influenza H3N2-resistant strains was associated with opposite clinical outcomes: 1 patient had mild upper respiratory syndrome; the other died of severe acute respiratory distress syndrome (*15*). On the other hand, on the basis of the observation that none of the patient’s family members and co-workers showed respiratory infection, we can hypothesize that immune impairment of the patient could have favored the zoonotic transmission of the SIV strain. Surveillance of circulating SIVs and monitoring of occupationally exposed workers are 2 important tools to prevent spread of potential pandemic viruses.

**Technical Appendix.** Phylogenetic tree based on polymerase bases 1 and 2, polymerase, nucleoprotein, matrix, and nonstructural gene sequences; and N-glycosylation predictions for the hemagglutinin proteins of the A/Brisbane/10/2007(H3N2), A/Pavia/07/2014(H3N2), and A/swine/Italy/282811/2013(H3N2) influenza strains.
