# Supplementary material for: Swine Influenza A(H3N2) Virus Infection in Immunocompromised Man, Italy, 2014
Source: Emerg Infect Dis. 2015 Jul;21(7):1189–91. doi: 10.3201/eid2107.140981 (PMC4480377; doi:10.3201/eid2107.140981)
Supplement: Supplementary file 1 — Technical Appendix. Phylogenetic tree based on polymerase bases 1 and 2, polymerase, nucleoprotein, matrix, and nonstructural gene sequences; and N-glycosylation predictions for the hemagglutinin proteins of the A/Brisbane/10/2007(H3N2), A/Pavia/07/2014(H3N2), and A/swine/Italy/282811/2013(H3N2) influenza strains. [file 14-0981-Techapp-s1.pdf]

Article DOI: http://dx.doi.org/10.3201/eid2107.140981

Swine Influenza A(H3N2) Virus Infection in Immunocompromised Man, Italy, 2014

Technical Appendix

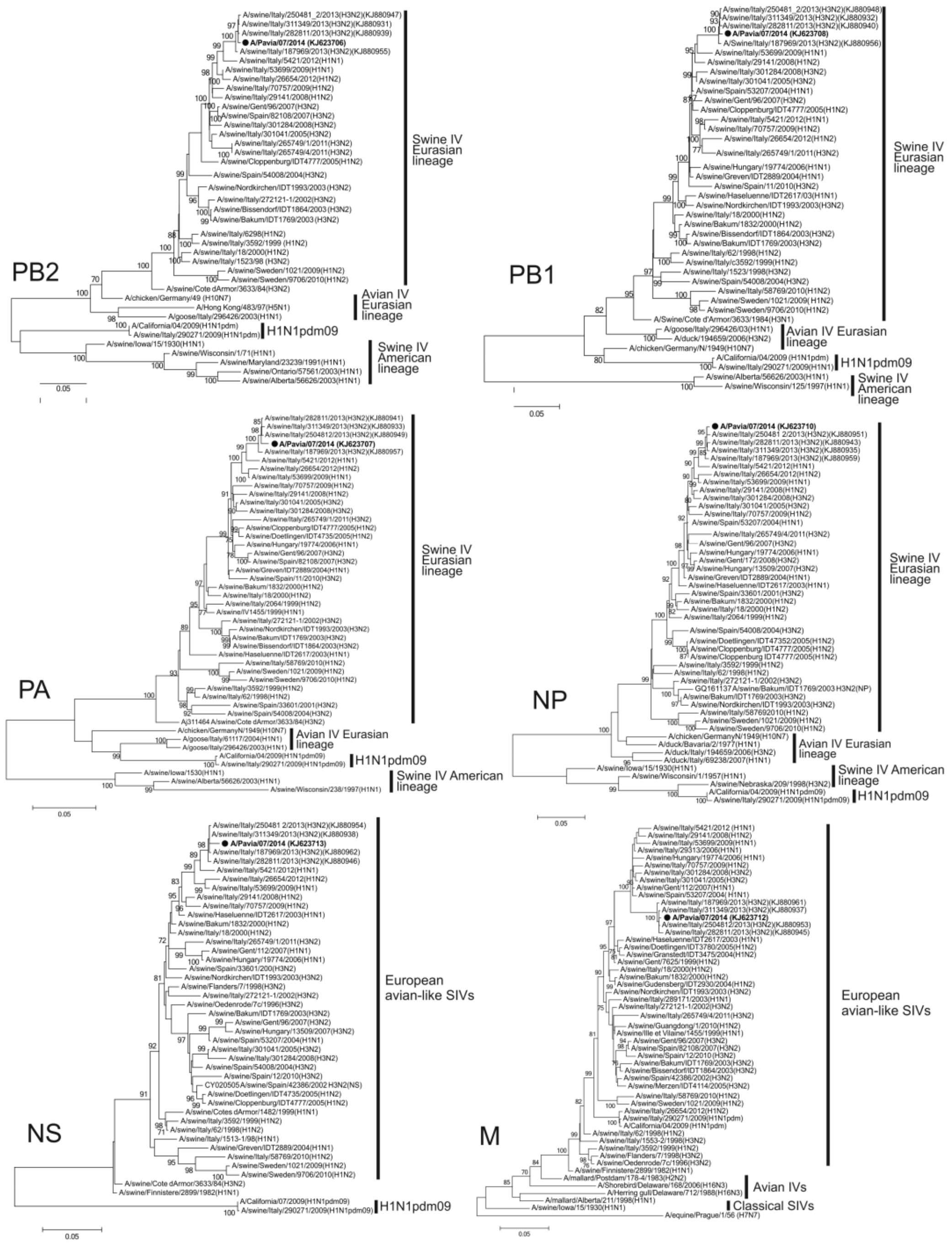

Technical Appendix Figure 1. Phylogenetic tree based on the polymerase base (PB) 1, PB2, polymerase (PA), nucleoprotein (NP), matrix (M), and nonstructural (NS) gene gene sequences.

A

```
Strain:  A/Brisbane/10/2007(H3N2)
MKTIIALSYILCLVFTQKLPGNDNSTATLCLGHHAVPNGTIVKTITNDQIEVTNATELVQSSSTGEICDSPHQILDGENC      80
TLIDALLGDPQCDGFQNKKWDLFVERSKAYSNCYPYDVPDYASLRSLVASSGTLEFNNESFNWTGVTQNGTSSACIRRSN     160
NSFFSRLNWLTHLKFKYPALNVTMPNNEKFDKLYIWGVHHPVTDNNQIFLYAQASGRITVSTKRSQQTVIPNIGSRPRVR     240
NIPSRISIYWTIVKPGDILLINSTGNLIAPRGYFKIRSGKSSIMRSDAPIGKCNSECITPNGSIPNDKPFQNVNRITYGA     320
CPRYVKQNTLKLATGMRNVPEKQTRGIFGAIAGFIENGWEGMVDGWYGFRHQNSEGIGQAADLKSTQAAIDQINGKLNRL     400
IGKTNEKFHQIEKEFSEVEGRIQDLEKYVEDTKIDLWSYNAELLVALENQHTIDLTDSEMNKLFEKTKKQLRENAEDMGN     480
GCFKIYHKCDNACIGSIRNGTYDHDVYRDEALNNRFQIKGVELKSGYKDWILWISFAISCFLLCVALLGFIMWACQKGNI     560
RCNICI
.......................N.............N........................................N.      80
.............................................................N......N...........     160
....................N...........................................................     240
.....................N......................................N...................     320
................................................................................     400
................................................................................     480
..................N.............................................................     560
......                                                                               640
```

(Threshold=0.5)

SeqName	Position		Potential	Jury agreement	N-Glyc result
A/Brisbane/10/2007(H3N2)	24	NSTA	0.8060	(9/9)	+++
A/Brisbane/10/2007(H3N2)	38	NGTI	0.6976	(9/9)	++
A/Brisbane/10/2007(H3N2)	54	NATE	0.4993	(3/9)	-
A/Brisbane/10/2007(H3N2)	79	NCTL	0.7092	(9/9)	++
A/Brisbane/10/2007(H3N2)	138	NESF	0.4296	(7/9)	-
A/Brisbane/10/2007(H3N2)	142	NWTG	0.5240	(6/9)	+
A/Brisbane/10/2007(H3N2)	149	NGTS	0.6570	(9/9)	++
A/Brisbane/10/2007(H3N2)	160	NNSF	0.4724	(7/9)	-
A/Brisbane/10/2007(H3N2)	181	NVTM	0.8151	(9/9)	+++
A/Brisbane/10/2007(H3N2)	262	NSTG	0.5879	(7/9)	+
A/Brisbane/10/2007(H3N2)	301	NGSI	0.6719	(9/9)	++
A/Brisbane/10/2007(H3N2)	499	NGTY	0.5203	(8/9)	+

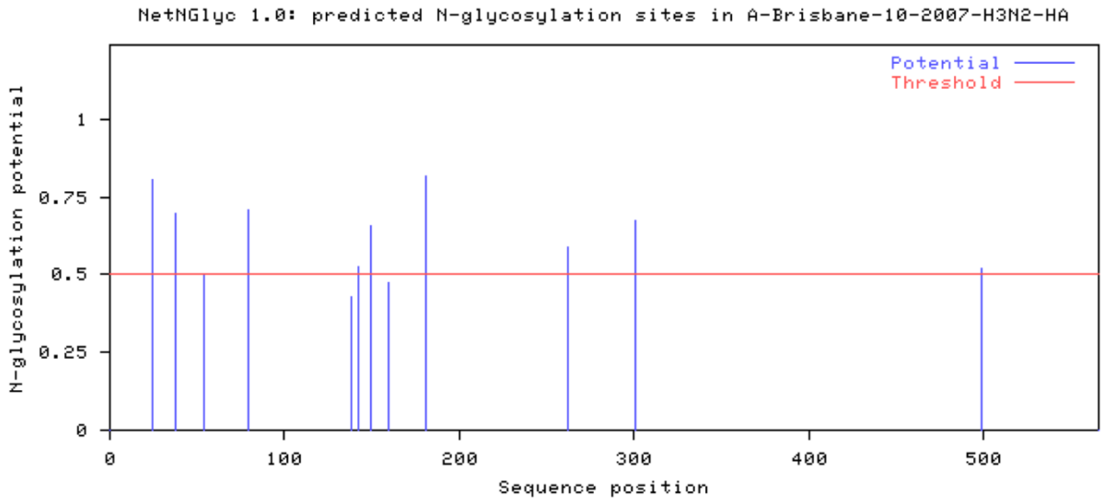

B

```
Strain: A/Pavia/07/2014(H3N2)
MKTVIALSYVFCLVFGQDFPGKGNNTATLCLGHHAVPNGTLVKTITDDQIEVTNATELVQNFSMGKICKNPHRILDGANC
TLIDSLLGDPHCDGFQNEKWDLFIERSRAFSNCYPYDVPEYTSLRSLIASSGTLEFTNENFNWTGVTQNGGSSACKRGPN
NSFFSRLNWLYKSGNTYPMLNVTMPNSDDFDKLYIWGVHHPSTDREQTNLYIQASGKIIVSTKRSQQTIIPNIGSRPWVR
GLSSRISIYWTIVKPGDILIINSNGNLIAPRGYFKIQTGKSSVMKSDAPIGTCNSECITPNGSIPNDKPFQNVNRITYGA
CPHYIKQSTLKLATGMRNIPERQTRGIFGAIAGFIENGWEGMVNGWYGFRHQNSEGIGQAADLKSTQAAINQINGKLNRV
IEKTNEKFHQIEKEFSEVEGRIQDLERYVEDTKIDLWSYNAELLVALENQHTIDLTDSEMNKLFEKTRKQLRENAEDMGN
GCFKIYHKCDNSCMESIRNGTYDHNEYRDEAVNNRFQIKSVELKSGYKDWILWISFAISCFLLCTIWMGFIIWACQKGNI
RCNICI
.......................N.............N......................N.................N.
.............................................................N..................
....................N...........................................................
............................................................N...................
................................................................................
................................................................................
..................N.............................................................
......
```

(Threshold=0.5)

SeqName	Position	Potential	Jury agreement	N-Glyc result
A/Pavia/07/2014(H3N2)	24 NNTA	0.7412	(9/9)	++
A/Pavia/07/2014(H3N2)	38 NGTL	0.7046	(9/9)	++
A/Pavia/07/2014(H3N2)	54 NATE	0.4940	(3/9)	-
A/Pavia/07/2014(H3N2)	61 NFSM	0.5011	(5/9)	+
A/Pavia/07/2014(H3N2)	79 NCTL	0.6842	(9/9)	++
A/Pavia/07/2014(H3N2)	142 NWTG	0.6148	(8/9)	+
A/Pavia/07/2014(H3N2)	160 NNSF	0.4433	(7/9)	-
A/Pavia/07/2014(H3N2)	181 NVTM	0.7646	(9/9)	+++
A/Pavia/07/2014(H3N2)	301 NGSI	0.6674	(9/9)	++
A/Pavia/07/2014(H3N2)	499 NGTY	0.6270	(9/9)	++

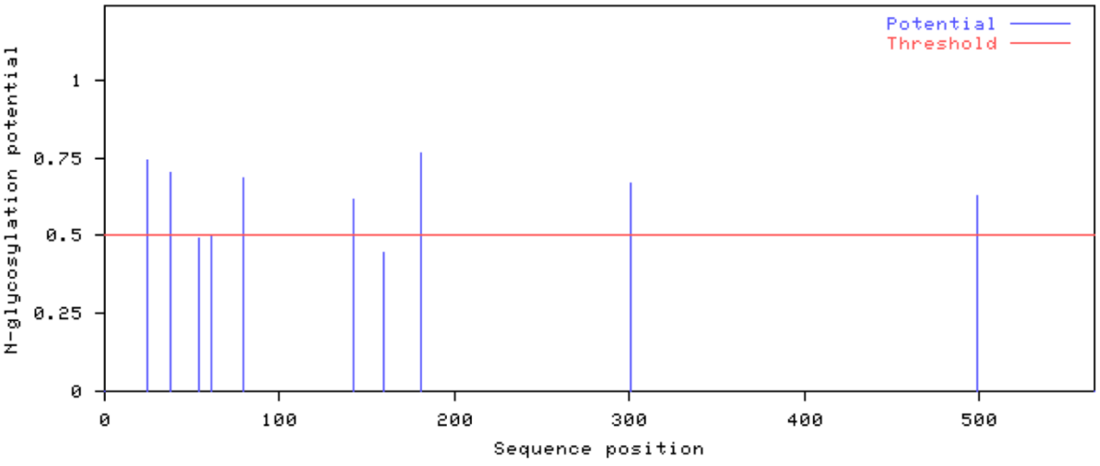

C

```
Strain: A/Swine/Italy/282811/2013(H3N2)
MKTVIALSYVFCLVFGQDFPGKGNNTATLCLGHHAVPNGTLVKTITDDQIEVTNATELVQNFSMGKICKNPHRILDGANC
TLIDSLLGDPHCDGFQNEKWDLFIERSRAFSNCYPYDVPEYTSLRSLIASSGTLEFTNENFNWTGVTQNGGSSACKRGPN
NSFFSRLNWLYKSGNTYPMLNVTMPNSDDFDKLYIWGVHHPSTDREQTNLYIQASGKIIVSTKRSQQTIIPNIGSRPWVR
GLSSRISIYWTIVKPGDILIINSNGNLIAPRGYFKIQTGKSSVMKSDAPIGTCNSECITPNGSIPNDKPFQNVNRITYGA
CPHYIKQNTLKLATGMRNIPERQTRGIFGAIAGFIENGWEGMVNGWYGFRHQNSEGIGQAADLKSTQAAINQINGKLNRV
IEKTNEKFHQIEKEFSEVEGRIQDLERYVEDTKIDLWSYNAELLVALENQHTIDLTDSEMNKLFEKTRKQLRENAEDMGN
GCFKIYHKCDNSCMESIRNGTYDHNEYRDEAVNNRFQIKSVELKSGYKDWILWISFAMSCFLLCAIWMGFIIWACQKGNI
RCNICI
.......................N.............N......................N.................N.
.............................................................N..................
....................N...........................................................
............................................................N...................
................................................................................
................................................................................
..................N.............................................................
......
```

(Threshold=0.5)

SeqName	Position		Potential	Jury agreement	N-Glyc result
A/Swine/Italy/282811/2013(H3N2)	24	NNTA	0.7412	(9/9)	++
A/Swine/Italy/282811/2013(H3N2)	38	NGTL	0.7046	(9/9)	++
A/Swine/Italy/282811/2013(H3N2)	54	NATE	0.4939	(3/9)	-
A/Swine/Italy/282811/2013(H3N2)	61	NFSM	0.5013	(5/9)	+
A/Swine/Italy/282811/2013(H3N2)	79	NCTL	0.6842	(9/9)	++
A/Swine/Italy/282811/2013(H3N2)	142	NWTG	0.6148	(8/9)	+
A/Swine/Italy/282811/2013(H3N2)	160	NNSF	0.4433	(7/9)	-
A/Swine/Italy/282811/2013(H3N2)	181	NVTM	0.7647	(9/9)	+++
A/Swine/Italy/282811/2013(H3N2)	301	NGSI	0.6673	(9/9)	++
A/Swine/Italy/282811/2013(H3N2)	499	NGTY	0.6271	(9/9)	++

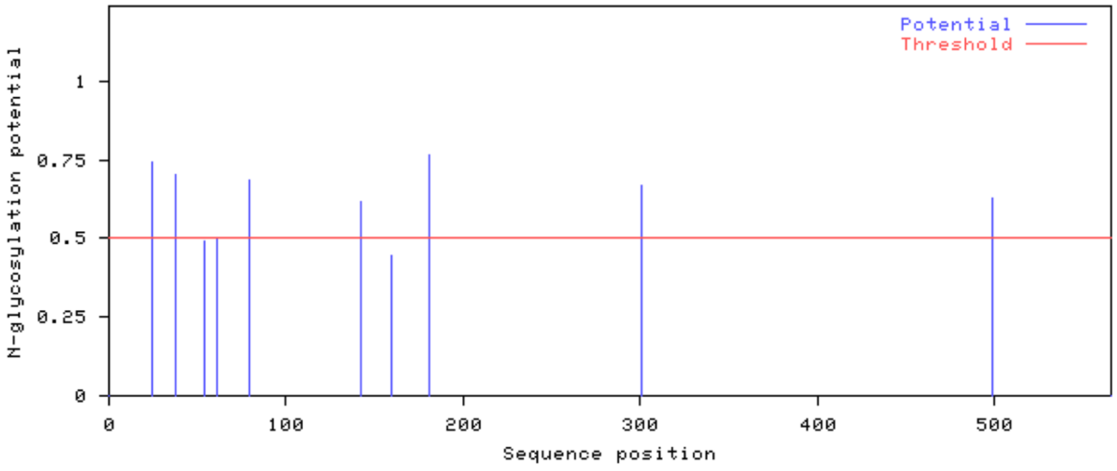

Technical Appendix Figure 2. N-glycosylation predictions for the hemagglutinin proteins of the A/Brisbane/10/2007(H3N2) (A), A/Pavia/07/2014(H3N2) (B), and A/Swine/Italy/282811/2013(H3N2) (C) influenza strains. Predictions were made by using the NetNGlyc 1.0 server available online (http://www.cbs.dtu.dk/services/NetNGlyc/). Asn-Xaa-Ser/Thr sequons in the sequence output are highlighted in blue. Asparagines predicted to be N-glycosylated are highlighted in red.
